# Supplementary material for: Oxidative Stress in Structural Valve Deterioration: A Longitudinal Clinical Study
Source: Biomolecules. 2022 Oct 31;12(11):1606. doi: 10.3390/biom12111606 (PMC9687638; doi:10.3390/biom12111606)
Supplement: Supplementary file 1 [file biomolecules-12-01606-s001.zip › biomolecules-1945696-supplementary.pdf]

Supplementary Material

Oxidative stress in structural valve deterioration: longitudinal clinical study

Short title: Oxidative stress in structural valve deterioration

Manuel Galiñanes[1,2*], Kelly Casós[1,3], Arnau Blasco-Lucas[1,2,4], Eduard Permanyer[1,2,5], Rafael Máñez[3], Thierry Le Tourneau[6,7], Jordi Barquinero[8], Simo Schwartz Jr[9], Tomaso Bottio[10], Jean Christian Roussel[11], Imen Fellah-Hebia[6], Thomas Sénage[6,12], Arturo Evangelista[13], Luigi P. Badano[14,15], Alejandro Ruiz-Majoral[16], Cesare Galli[17], Vered Padler-Karavani[18], Jean-Paul Soulillou[19], Xavier Vidal[20], Emanuele Cozzi[21], Cristina Costa[3].

[1]Reparative Therapy of the Heart, Vall d'Hebron Research Institute (VHIR), Autonomous University of Barcelona (UAB), Barcelona, Spain
[2]Department of Cardiac Surgery, University Hospital Vall d'Hebron (HUVH)-ICS, Barcelona, Spain
[3]Infectious Diseases and Transplantation Division, Bellvitge Biomedical Research Institute (IDIBELL) and Bellvitge University Hospital-ICS, L'Hospitalet de Llobregat, Barcelona, Spain
[4]Department of Cardiac Surgery, Bellvitge University Hospital-ICS, L'Hospitalet de Llobregat, Barcelona, Spain
[5]Department of Cardiac Surgery, Quironsalud Teknon Heart Institute, Barcelona, Spain
[6]CHU Nantes, Université de Nantes, Institut du Thorax, CIC 1413, F-44000 Nantes, France
[7]Université de Nantes, CHU de Nantes, CNRS, INSERM, Institut du Thorax, F-44000 Nantes, France
[8]Gene and Cell Therapy, VHIR, UAB, Barcelona, Spain
[9]Department of Nanotechnology (CIBBIM), VHIR, UAB, Barcelona, Spain
[10]Department of Cardio Thorac Vascular Sciences and Public Health, Padua University Hospital, Padua, Italy
[11]Department of Thoracic and Cardiovascular Surgery, Institut du Thorax, University Hospital, Nantes, France
[12]Biostatistic Department, UNIV Nantes, Nantes, France
[13]Department of Cardiology, Vall d´Hebron Research Institut (VHIR), Hospital Vall d´Hebron, Barcelona, Spain
[14]Department of Medicine and Surgery, University of Milano-Bicocca, Milan, Italy
[15]Department of Cardiology, Neural and Metabolic Sciences, Istituto Auxologico Italiano, IRCCS, San Luca Hospital, Milan, Italy
[16]Department of Cardiology, Bellvitge University Hospital, L'Hospitalet de Llobregat, Barcelona, Spain;
[17]Avantea and Fondazione Avantea Onlus, 26100 Cremona, Italy
[18]Department of Cell Research and Immunology, The Shmunis School of Biomedicine and Cancer Research, The George S. Wise Faculty of Life Sciences, Tel Aviv University, Tel Aviv, 69978, Israel
[19]Institut de Transplantation–Urologie–Néphrologie, INSERM Unité Mixte de Recherche 1064, Centre Hospitalier Universitaire de Nantes, Nantes, France;
[20]Department of Clinical Pharmacology, HUVH-ICS, Barcelona, Spain
[21]Transplant Immunology Unit, Department of Transfusion Medicine, Padua University Hospital, Padua, Italy.

[*]Correspondence to:

Manuel Galiñanes MD, PhD.
e-mail: manuel.galinanes@gmail.com; telephone number: +34 609 829 170

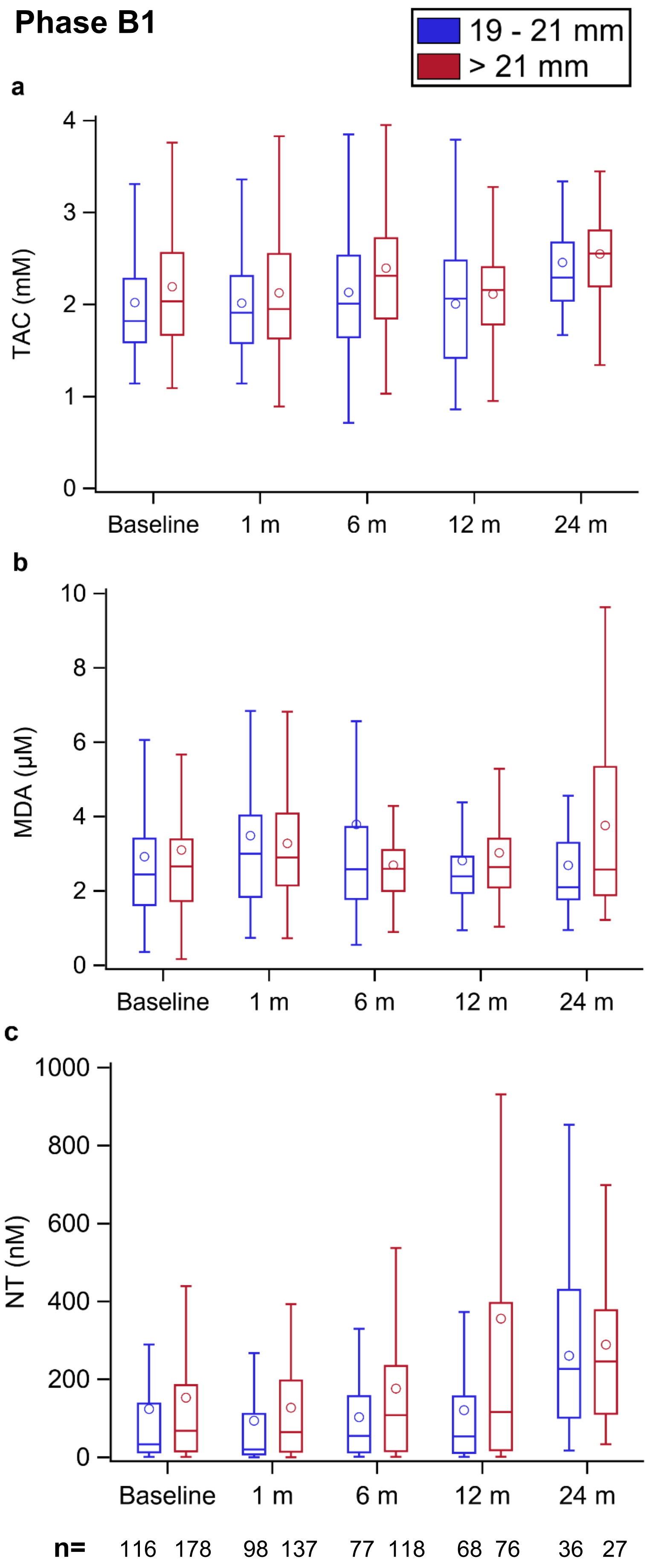
Phase B1
19 - 21 mm
> 21 mm
a
TAC (mM)
0
1
2
3
4
Baseline
1 m
6 m
12 m
24 m
b
MDA (µM)
0
2
4
6
8
10
Baseline
1 m
6 m
12 m
24 m
c
NT (nM)
0
200
400
600
800
1000
Baseline
1 m
6 m
12 m
24 m
n=
116 178
98 137
77 118
68 76
36 27

Supplementary Figure S1: (a) Total antioxidant capacity (TAC, in mM), (b) malondialdehyde (MDA, in *μ*M), and (c) nitrotyrosine (NT, in nM) values in Phase B1 with implanted aortic biological heart valves (BHVs) according to their size (19-21 mm diameter in blue boxplots and >21 mm diameter in red boxplots) at different time points (baseline and 1, 6, 12 and 24 months after implantation). The numbers at the bottom of the figures represent the number of assessed patients. There were no significant statistical differences between the two study groups at any time-point.

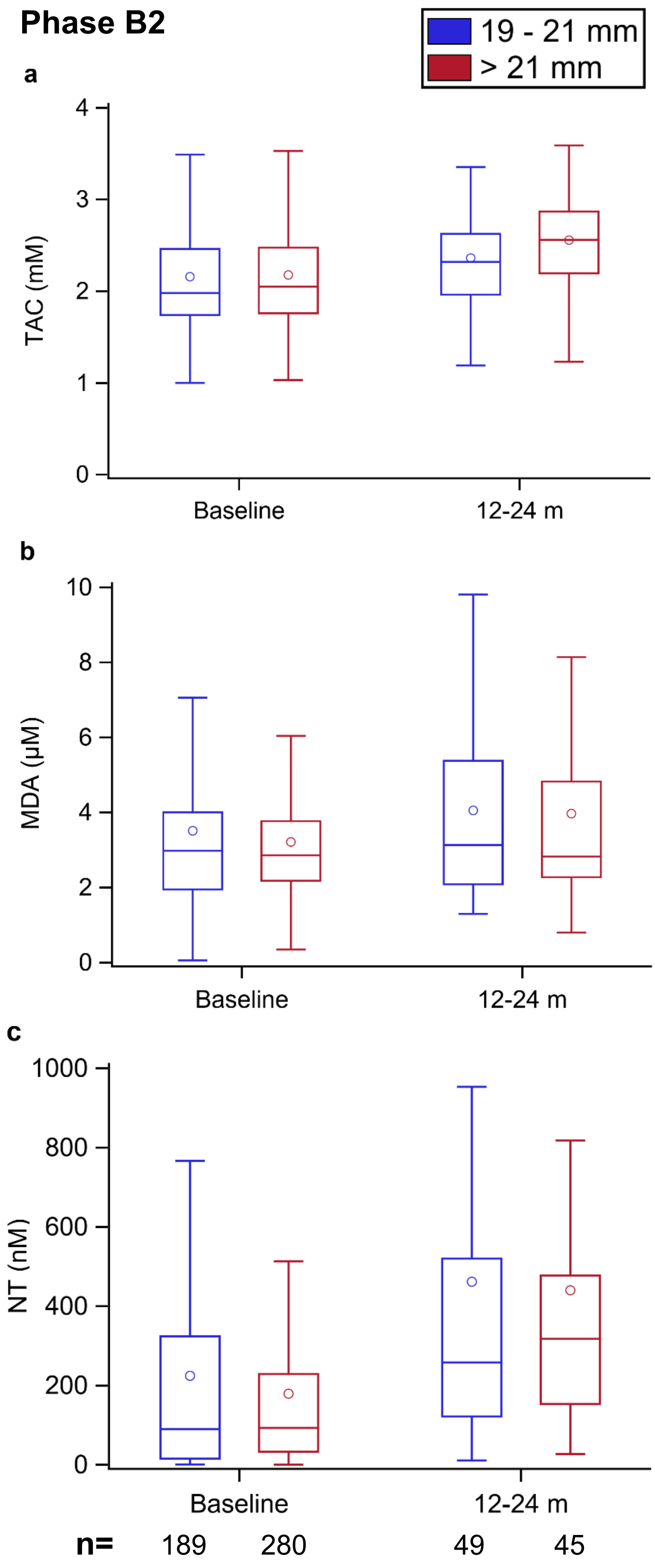

Phase B2
19 - 21 mm
> 21 mm
a
TAC (mM)
4
3
2
1
0
Baseline
12-24 m
b
MDA (μM)
10
8
6
4
2
0
Baseline
12-24 m
c
NT (nM)
1000
800
600
400
200
0
Baseline
12-24 m
n=
189
280
49
45

Supplementary Figure S2: (a) Total antioxidant capacity (TAC, in mM), (b) malondialdehyde (MDA, in μM), and (c) nitrotyrosine (NT, in nM) values in Phase B2 patients with implanted aortic biological heart valves (BHVs) at the time of recruitment (>48 months after aortic valve replacement) and after 12-24 month follow-up (>60-72 months after aortic valve implantation) according to their size (19-21 mm diameter in blue boxplots and >21 mm diameter in red boxplots). The numbers at the bottom of the figures represent the number of assessed patients. There were no statistical significant differences between the two study groups at the two time-points.

SUPPLEMENTARY TABLE S1. Types of mechanical prostheses implanted.

**Type of prosthesis**	**Phase B1** (n=37)	**Phase B2** (n=36)
ATS	9	2
Carbomedics (Standard/Carbo-Seal/Top Hat)	9 (7/2/0)	20 (18/0/2)
Sorin Bicarbon (Fitline/Overline)	15 (14/1)	7 (5/2)
St Jude Medical (Standard/Regent)	4 (1/3)	7 (6/1)

SUPPLEMENTARY TABLE S2. Time to detection (months) of structural valve deterioration (SVD) in implanted bioprostheses.

	n	Mitroflow	n	Perimount	n	Other pericardium bioprostheses	n	Porcine aortic valve
Stenosis (range/mean[SD])	43	35.9-130/81.3 (22.4)	23	48.9-261/120.3 (53.4)	1	63.8-63.8/63.8 (-)	9	32-136.5/89.7 (29.1)
19-21 mmHg	35	35.9-130/77.4 (22.0)	11	62.6-189/107.8 (39.8)	0	-	2	72.1-72.9/72.5 (0.6)
> 21 mmHg	8	75.6-120.3/98.4 (15.6)	12	48.9-261/131.8 (62.9)	1	63.8-63.8/63.8 (-)	7	32-136.5/94.6 (31.6)
Regurgitation* (range/mean[SD])	7	64.9-114.7/89.6 (18.5)	0	-	0	-	2	123.5-151.2/137.4 (19.6)
Mixed stenosis + regurgitation (range/mean[SD])	26	64.8-143/98.8 (19.6)	10	60.2-229.1/162.1 (49.3)	3	39.1-54.6/48.2 (8.1)	4	103.2-217.5/147.7 (50.7)
19-21 mmHg	18	64.8-143/104.8 (19.2)	3	60.2-176.8/133.5 (63.9)	2	39.1-51/45.1 (8.4)	2	118.4-151.5/135 (23.4)
> 21 mmHg	7	67.9-106.3/84.7 (14.3)	6	127.3-229.1/170.7 (43.9)	1	54.6-54.6/54.6 (-)	2	103.2-217.5/160.4 (80.8)

* All valves > 21 mmHg

SUPPLEMENTARY TABLE S3. Effect (P values) of comorbidities on oxidative stress >48 months after BHV implantation (Phase B2).

Comorbid condition		n	TAC (mM)	P value	MDA (µM)	P value	NT (nM)	P value
Diabetes mellitus	Yes	132	2.14 (2.05-2.24)	0.328	2.94 (2.61-3.30)	0.540	73.5 (55.7-97.0)	0.993
	No	342	2.08 (2.03-2.14)		2.82 (2.68-2.98)		73.6 (61.5-88.1)	
Arterial hypertension	Yes	400	2.09 (2.03-2.14)	0.494	2.93 (2.78-3.09)	0.054	73.0 (61.8-86.3)	0.827
	No	71	2.14 (2.01-2.27)		2.49 (2.13-2.92)		69.7 (48.8-99.5)	
Obesity	Yes	159	2.11 (2.03-2.21)	0.654	2.91 (2.69-3.16)	0.584	64.9 (49.5-85.0)	0.239
	No	317	2.09 (2.03-2.15)		2.83 (2.65-3.01)		78.5 (65.6-93.9)	
Ischemic heart disease	Yes	36	2.23 (1.97-2.53)	0.285	3.21 (2.60-3.97)	0.188	128.9 (94.5-175.9)	0.001
	No	436	2.09 (2.04-2.14)		2.83 (2.69-2.98)		71.1 (60.6-83.4)	

BHV, bioprosthetic heart valve; MDA, malondialdehyde; NT, nitrotyrosine; TAC, total antioxidant capacity
Values are presented as geometric mean (95% lower limit of mean – 95% upper limit of mean)

SUPPLEMENTARY TABLE S4. Effect (P values) of treatment with metformin on oxidative stress in BHV patients in Phase B1.

Time course	Treatment with Metformin	n	TAC (mM)	P value	MDA (μM)	P value	NT (nM)	P value
Baseline	Yes	57	1.85 (1.73-1.99)	0.017	2.16 (1.78-2.62)	0.083	27.8 (17.6-43.9)	0.006
	No	244	2.06 (1.98-2.14)		2.50 (2.30-2.72)		53.7 (44.0-65.7)	
1 m follow-up	Yes	46	2.01 (1.88-2.15)	0.665	2.53 (2.16-2.96)	0.118	15.6 (10.1-24.3)	<0.001
	No	193	1.98 (1.90-2.01)		2.99 (2.77-3.23)		44.4 (34.6-57.04)	
6 m follow-up	Yes	39	2.13 (1.95-2.31)	0.400	2.54 (2.14-3.00)	0.789	21.2 (12.3-36.7)	<0.001
	No	159	2.21 (2.10-2.32)		2.63 (2.42-2.87)		69.4 (54.6-88.3)	
12 m follow-up	Yes	36	2.07 (1.87-2.30)	0.802	2.90 (2.52-3.34)	0.267	56.8 (29.9-108.1)	0.173
	No	112	1.96 (1.84-2.08)		2.55 (2.35-2.77)		62.5 (44.2-88.4)	
24 m follow-up	Yes	20	2.48 (2.30-2.68)	0.854	2.37 (1.89-2.98)	0.335	186.4 (113.9-304.9)	0.078
	No	44	2.40 (2.22-2.60)		2.73 (1.89-2.98)		188.9 (142.8-249.8)	

BHV, bioprosthetic heart valve; MDA, malondialdehyde; NT, nitrotyrosine; TAC, total antioxidant capacity
Values are presented as geometric mean (95% lower limit of mean – 95% upper limit of mean)

SUPPLEMENTARY TABLE S5. Effect (P values) of treatment with metformin on oxidative stress in BHV patients in Phase B2 and Phase A (patients with SVD).

	Treatment with Metformin	n	TAC (mM)	P value	MDA (μM)	P value	NT (nM)	P value
**Phase B2**								
Baseline	Yes	69	1.97 (1.87-2.09)	0.041	2.85 (2.42-3.36)	0.948	57.0 (37.9-85.7)	0.155
	No	407	2.12 (2.07-2.18)		2.86 (2.72-3.01)		76.4 (65.0-89.8)	
12-24 m follow-up	Yes	13	2.52 (2.23-2.85)	0.347	3.65 (2.53-5.26)	0.492	412.3 (260.0-653.9)	0.272
	No	83	2.35 (2.21-2.49)		3.25 (2.85-3.70)		245.7 (192.9-312.8)	
**Phase A**	Yes	14	2.31 (2.01-2.65)	0.244	2.55 (1.69-3.83)	0.730	32.3 (15.2-68.5)	0.079
	No	98	2.08 (1.96-2.22)		2.41 (2.15-2.69)		70.4 (51.4-96.5)	

BHV, bioprosthetic heart valve; MDA, malondialdehyde; NT, nitrotyrosine; SVD, structural valve deterioration; TAC, total antioxidant capacity
Values are presented as geometric mean (lower limit of mean – upper limit of mean).
